# Selected Properties of Composite Materials Used for Dental Fillings—Methodological Development and Preliminary Results

**DOI:** 10.3390/ma19010146

**Published:** 2025-12-31

**Authors:** Katarzyna Piotrowska, Monika Madej, Joanna Wysokińska-Miszczuk, Michał Paulo

**Affiliations:** 1 Faculty of Mechatronics and Mechanical Engineering, Kielce University of Technology, al. Tysiąclecia Państwa Polskiego 7, 25-314 Kielce, Poland; mmadej@tu.kielce.pl; 2 Faculty of Medical Dentistry, Medical University of Lublin, ul. Chodźki 6 Ave, 20-093 Lublin, Poland; joanna.wysokinska-miszczuk@umlub.pl (J.W.-M.); michal.paulo@umlub.pl (M.P.)

**Keywords:** dental composites, morphology, hardness, wettability, friction

## Abstract

Dental composites are widely used in restorative dentistry; however, their long-term clinical performance is strongly influenced by mechanical and tribological behavior under oral conditions. Understanding the relationship between material structure, surface characteristics, and functional properties is therefore essential. This preliminary methodological study evaluated the mechanical, tribological, and wetting properties of three light-cured dental composites—Enamel Plus HRi, Amaris, and Estelite Asteria—commonly used in clinical practice. The materials were characterized in terms of surface morphology, hardness, Young’s modulus, coefficient of friction, and wear resistance under controlled laboratory conditions. Instrumental indentation and tribological tests were performed, and results were expressed as mean values with standard deviations calculated from multiple measurements. The results demonstrated that filler composition and surface topography affected material performance. Estelite Asteria exhibited the highest hardness (HIT > 300 MPa), while Enamel Plus HRi showed the highest Young’s modulus (EIT ≈ 14.5 GPa). Materials with more complex surface morphology retained lubricating artificial saliva more effectively, resulting in lower friction coefficients (minimum µ = 0.85), although this did not reduce wear. The highest wear was observed for Estelite Asteria, with a wear scar approximately 62% greater than that of Enamel Plus HRi. These preliminary findings provide a methodological basis for further investigations under more clinically relevant conditions.

## 1. Introduction

Composite resin restorations constitute the most commonly used solution in the conservative treatment of permanent teeth—both for the restoration of carious and non-carious lesions, as well as for esthetic and functional reconstructions of worn dentition. Given their widespread clinical use and the requirements concerning durability, esthetics, biocompatibility, adhesion, and wear resistance, the physicochemical and technological properties of these materials are of considerable clinical and research relevance. In dental practice, the selection of an appropriate material must account for ease of application, compatibility with the type of cavity, mechanical strength, and aesthetic and polishing properties. The ability to achieve adequate surface smoothness and gloss, as well as the tribological performance of the material, are also important considerations. Moreover, the material must exhibit high resistance to the conditions prevailing in the oral environment, including mechanical loading, abrasion, moisture exposure, interaction with oral fluids, and temperature fluctuations [1,2,3,4,5,6,7].

Due to their favorable physical and mechanical properties, composite materials are used not only in restorative dentistry but also in prosthodontics, where they serve for reshaping teeth or performing complete reconstructions as uniform fixed or temporary restorations [8,9,10,11]. In patients with pathological tooth wear, these materials are employed to restore the appropriate occlusal vertical dimension. Moreover, composites have broad applications in other fields of dentistry, including splinting teeth after trauma, stabilizing teeth with pathological mobility in periodontics, and cementing components of fixed orthodontic appliances [12,13].

The literature distinguishes several groups of resin-based restorative materials, including flowable composites (low-viscosity or semi-fluid composites) and conventional composites (hybrid, mini-hybrid, micro-hybrid, and related types), as well as ormocers, which represent a combination of composite resin technology with ceramic components. Flowable materials are characterized by lower viscosity, reduced filler content, and easier application in hard-to-reach areas of the cavity or as a lining layer [1,14,15,16]. The conventional composites—due to their higher filler content, greater viscosity, and typically more advanced technology—are widely used for the restoration of both anterior and posterior teeth, where favorable mechanical properties, color stability, and esthetic quality are required [17,18]. In a review [19], clinical outcomes of flowable and conventional composites were compared. It was found that despite differences in composition and physicochemical properties, no differences in clinical effectiveness between flowable and conventional composites were observed during short-term follow-up (up to 36 months). The authors, however, emphasized that factors such as occlusal conditions, material layer thickness, cavity location, and observation period may influence the results. These findings are supported by eight-year clinical studies, which demonstrated higher wear resistance for conventional composites compared with flowable materials in the restoration of cervical lesions [20].

The long-term exposure of composite materials in the oral environment means that their proper function depends not only on adhesive conditions and material properties but also on parameters such as surface topography, wettability, and tribological resistance. Therefore, this article presents the results of model studies of conventional composite materials, including the evaluation of the above-mentioned parameters to assess their potential clinical durability in the oral environment. Accordingly, the present study was designed to systematically evaluate these parameters, providing insights into the mechanical behavior, frictional performance, and wear resistance of these commonly used light-cured composites, and thereby assessing their potential clinical durability.

## 2. Materials and Methods

### 2.1. Materials

Three composite materials were selected for this study—Enamel Plus HRi (I), Amaris (II), and Estelite Asteria (III) (Table 1)—which represent the most commonly described groups of contemporary restorative composites (microhybrid, nanohybrid, and supra-nanohybrid). Conventional composites provide superior mechanical strength, color stability, and high esthetic quality after polishing. The selection of these materials was motivated by their widespread clinical use in routine dental practice, as well as their diverse chemical composition and physicochemical properties, which allow for comparison in terms of selected functional parameters. Moreover, they represent different groups of modern light-cured composites currently available on the market, enabling a more comprehensive assessment of their behavior under conditions simulating the oral environment.

The materials tested were conventional composites, commonly used for the restoration of both anterior and posterior teeth. They are characterized by favorable mechanical properties, color stability, and high surface esthetics after polishing [21,22,23]. The fundamental information regarding the material parameters, including the resin matrix (monomers), filler content (wt%), and filler type and size, is summarized in Table 2. All information on filler type, size, and content was obtained from manufacturers’ technical data sheets.

Composite disks with a diameter of 12 mm and a thickness of 2.5 mm were fabricated. Owing to its design and integrated scale, the device used for specimen preparation allowed precise control and measurement of the thickness of each applied layer—achieved by rotating the lower part of the mold—until the final dimensions of the specimen were obtained. The specimens were prepared by a single operator in accordance with the manufacturer’s instructions. The composite was applied incrementally using a PIF applicator (Megadenta, Radeberg, Germany); each 0.5 mm layer was condensed and subsequently smoothed with the applicator to ensure a uniform surface. Each increment was light-cured from a distance of less than 1 mm using a Megalux LED lamp (Megadenta, Radeberg, Germany) in full-power mode for 20 s at an intensity of 1200 mW/cm^2^. Rotation of the device’s adjustment mechanism created space for the next increment. The finished specimens were not subjected to any mechanical treatment—neither polishing with rubber nor smoothing with dental burs. Surface smoothness was achieved solely through the application and condensation procedure. Before testing, all specimens were stored in a desiccator under controlled environmental conditions at a temperature of 23 °C and a relative humidity of 55%. The results presented constitute a preliminary stage of the research; the next stage will involve the analysis of mechanically treated specimens polished using various techniques.

### 2.2. Methods

The surface morphology of the tested composite materials was examined using a Phenom XL scanning electron microscope (Thermo Fisher Scientific, Eindhoven, The Netherlands). Disk-shaped specimens with a diameter of 12 mm were analyzed using a secondary electron detector (SED) under vacuum conditions of 1 Pa, with an accelerating voltage of 15 kV and a magnification of up to ×3000. The results are presented in Section 3.1.

Instrumental hardness measurements were performed using a UNHT nanoindenter (Anton Paar, Baden, Switzerland) equipped with a Berkovich diamond indenter with a tip radius of approximately 100 nm. The tests were conducted following the Oliver and Pharr method, with a loading and unloading rate of 100 mN/min, a maximum load of 50 mN, and a pause of 5 s. For each material, 10 independent measurement series were performed in accordance with the relevant standard [24], and the results are presented in Section 3.2.

Wettability was evaluated using an Attension Theta Flex tensiometer (Biolin Scientific, Tietäjänte, Finland) employing the sessile drop method. Artificial saliva was used as the test liquid, and the drop volume was 4 µL. The mean contact angle was calculated from 10 independent measurements. The tests were conducted in accordance with the relevant standard [25], and the results are presented in Section 3.3.

Tribological tests were carried out using a TRB tribometer (Anton Paar, Baden, Switzerland). The experiments were designed as model tribological investigations, aimed at evaluating the preliminary friction and wear behavior of the tested materials. Disk-shaped specimens with a diameter of 12 mm were tested under reciprocating motion with a normal load of 1 N, an amplitude of 5 mm, and a frequency of 1 Hz for a total of 10,000 cycles. A ZrO_2_ ball with a diameter of 6 mm was used as the counter-sample. All tests were conducted in artificial saliva at 37 °C. For each material, three independent measurement series were performed to ensure repeatability of the results. The chemical composition of the artificial saliva used in the tribological tests is provided in Table 3.

Hertzian contact pressures were calculated for the point contact between the ZrO_2_ sphere and the composite surfaces, and the maximum pressures were found to be 168 MPa, 123 MPa, and 156 MPa for Enamel Plus HRi, Amaris, and Estelite Asteria, respectively. After the tribological tests, the surface texture was assessed using a DCM8 non-contact optical profilometer (Leica, Heerbrugg, Switzerland). Measurements were performed in confocal mode using a ×20 objective lens, with a scanned area of 1.2 mm × 1.2 mm. Three independent surface topography measurements were carried out for each specimen. Based on these observations, the average values of the maximum wear track depth and wear track area were determined. The results are presented in Section 3.4.

## 3. Results

### 3.1. Morphology

**Figure 1:**
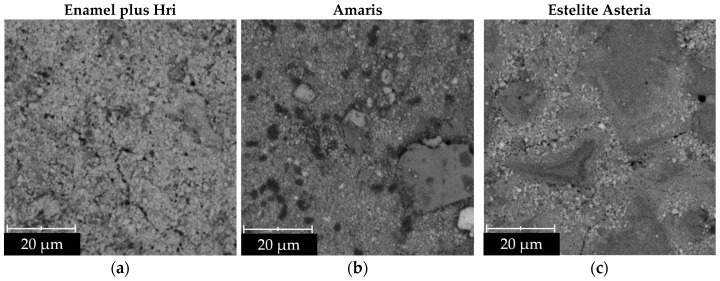
Representative SEM images of dental materials: Enamel plus Hri (**a**), Amaris (**b**), and Estelite Asteria (**c**).

Surface morphology analysis was performed using SEM micrographs. Representative SEM images are shown in Figure 1.

**Figure 2:**
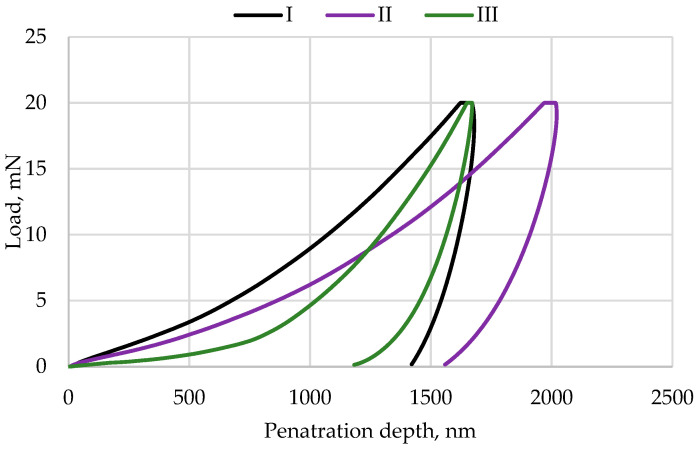
Representative graphs of loading–unloading curves as a function of indenter penetration depth: Enamel plus Hri (I), Amaris (II), and Estelite Asteria (III).

The test results presented in Figure 2 indicate that materials II and III showed the most complex geometric surface structure. The SEM images revealed the presence of large filler particles, either larger in size or with more complex morphology. In specimen II, micrometer-sized barium–alumino–borosilicate glass grains and prepolymer particles were observed, whereas specimen III contained spherical silica–zirconia fillers with a high weight fraction. Due to the absence of any finishing treatment, these particles remained clearly visible after polymerization. Enamel Plus HRi (I) exhibited a moderately developed surface topography, which was associated with its nanohybrid filler system comprising both nanoparticles and glass microparticles.

Literature in the fields of tribology, materials science, and dentistry clearly demonstrates that surface morphology influences frictional behavior, wettability, mechanical performance, and wear resistance [27,28,29,30].

### 3.2. Hardness

Instrumental indentation was used to evaluate the mechanical properties of the tested composite materials. These measurements allowed for precise recording of the correlation between applied load and penetration depth at the nanoscale (Figure 2). Based on the recorded load–displacement curves, two key material characteristics were determined: instrumented hardness (H_IT_), reflecting resistance to permanent plastic deformation, and Young’s modulus (E_IT_), representing the material’s elasticity. The indentation points were selected randomly across the sample surface to avoid local bias related to material heterogeneity. At the same time, appropriate spacing between adjacent indentations was maintained to prevent interaction of plastic deformation zones. Additionally, all indentation points were positioned at a sufficient distance from the specimen edges, exceeding 2.5 times the indentation size, to avoid edge effects. This approach enabled a comprehensive comparison of the tested composites in terms of their mechanical properties. Table 4 presents the average values of these parameters obtained from 10 measurement series.

The results of the mechanical tests indicate that Estelite Asteria (III) showed the highest instrumented hardness among the tested materials, which can be attributed to its very high content of hard silica–zirconia fillers (82 wt%). This finding suggests that this composite has the potential to demonstrate the greatest wear resistance. For the nanohybrid composite Enamel Plus HRi (I), the highest Young’s modulus was recorded, indicating the greatest stiffness among the examined materials. This behavior is associated with the presence of both zirconia nanoparticles and glass microparticles within the filler system. Material II (Amaris) showed the lowest hardness and Young’s modulus values, reflecting its moderate filler content and the characteristics typical of flowable–hybrid composite formulations. Overall, the results clearly demonstrate a strong dependence of mechanical performance on composite composition, particularly the type and proportion of filler and the properties of the resin matrix. Materials with higher contents of hard fillers generally exhibit greater hardness and elastic modulus [28,29]. The observed differences in hardness and stiffness may have important implications for clinical performance, including wear resistance, susceptibility to deformation under functional loading, and long-term durability.

### 3.3. Contact Angle

**Figure 3:**
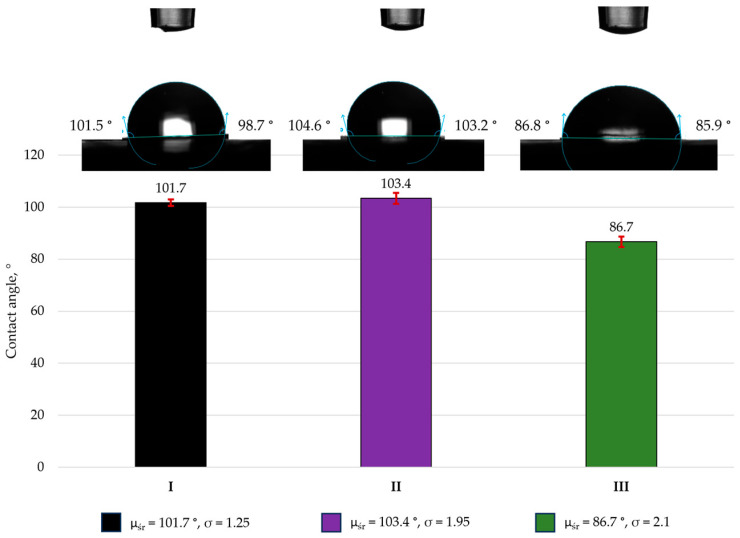
Average values of the contact angle with artificial saliva at pH 6.8 of dental materials: Enamel plus Hri (I), Amaris (II), and Estelite Asteria (III).

For the assessment of wettability, the specimens were placed on the measurement table, and a droplet of the test liquid was dispensed onto the surface. The contact angle formed at the liquid—solid interface was then recorded. A photo of the drop was taken with a digital camera 2 s after the drop was deposited on the sample surface. To ensure reliability, the mean contact angle was calculated from 10 independent measurements. The obtained data are summarized in Figure 3.

**Figure 4:**
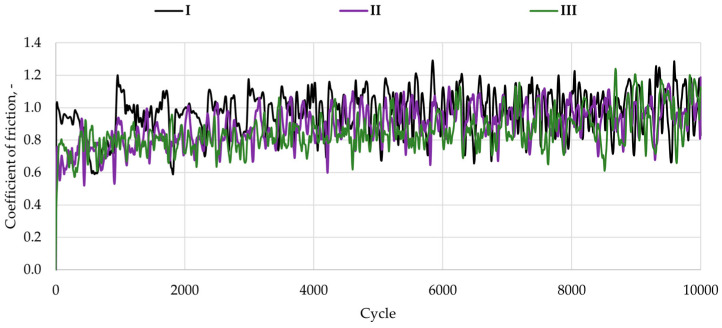
Example waveforms of friction coefficients: Enamel plus Hri (I), Amaris (II), and Estelite Asteria (III).

The results presented in Figure 4 indicate that surface morphology influenced the contact angle values. The lowest contact angle with artificial saliva—86.7°—was recorded for Estelite Asteria (III), which exhibited the most complex surface topography. This value was approximately 15% lower than that of Enamel Plus HRi (I) and Amaris (II). For the remaining samples (I and II), the contact angle exceeded 90°, indicating hydrophobic behavior. From a clinical perspective, hydrophobicity is desirable in dental restorations [31]. In the oral environment, both natural tooth tissues and composite restorations are continuously exposed to saliva. Because caries is a bacterial disease, microorganisms readily migrate and colonize both dental tissues and restorative surfaces. When a material demonstrates hydrophilic behavior, the integrity of the tooth–restoration interface may be compromised, increasing the risk of microleakage. This issue is particularly relevant in areas that are difficult to maintain, such as class II and class V cavities, where microleakage promotes secondary caries formation. Within the oral cavity, each restoration is continuously exposed to saliva, and interactions between restorative materials and the surrounding environment can influence their long-term performance. Increased hydrophilicity of composite materials promotes staining both at the restoration–tooth interface and on the restoration surface itself. Consequently, the esthetic quality of the restoration is reduced, which often leads to decreased patient satisfaction with the treatment outcome [31,32,33].

### 3.4. Tribological Tests and Assessment of Surface Texture After Tribological Tests

**Figure 5:**
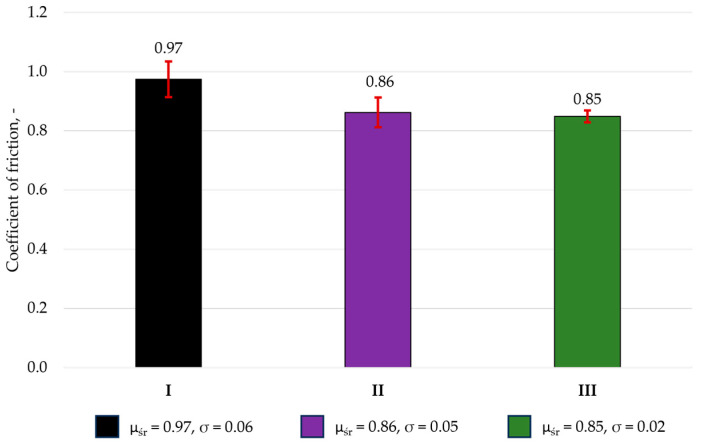
Average values of friction coefficients: Enamel plus Hri (I), Amaris (II), and Estelite Asteria (III).

The purpose of the friction–wear tests was to determine the coefficients of friction and the volumetric index wear of the tested pairs. Figure 4 presents representative friction coefficient curves, while Figure 5 shows the average friction coefficient values, calculated from three measurement series.

The results indicate that all analyzed materials exhibited similar coefficients of friction (µ), ranging from 0.85 to 0.97. The lowest coefficients of friction were observed for specimens with the most complex surface topography, namely Amaris (0.86) and Estelite Asteria (0.85). This is likely due to the retention of the lubricating medium—artificial saliva—in surface valleys, which promotes the formation of a lubricating film and reduces friction within the contact pair, as reported previously for dental resin composites tested under lubricated conditions. The highest friction coefficients were recorded for the specimens with moderately developed surface geometry (Enamel Plus HRi). It was also observed that composites with the lowest surface roughness exhibited similarly high µ values, which may be attributed to the limited retention of the lubricating fluid. Similar observations were reported by [34]. The authors demonstrated that filler structure and surface topography have a direct impact on the tribological properties of composites used in dental restorations.

**Figure 6:**
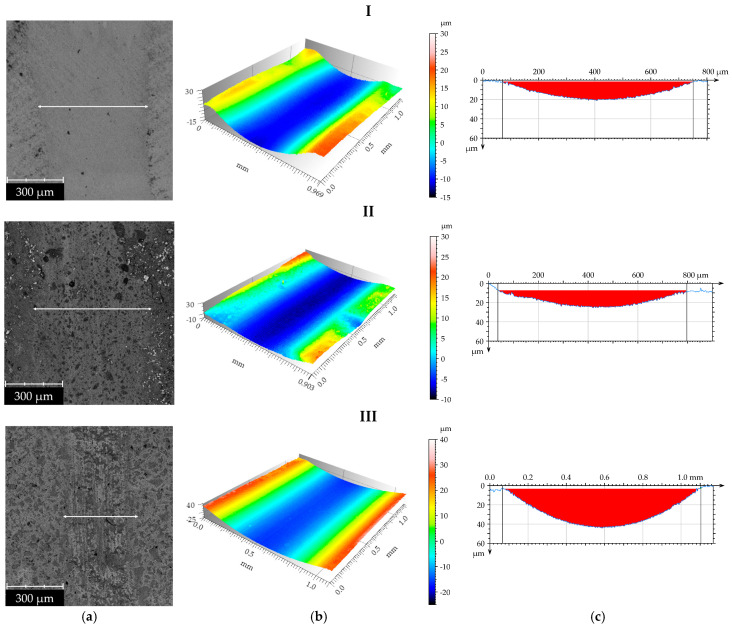
Surface texture after tribological tests: Representative SEM images (**a**), three-dimensional axonometric views (**b**), and profiles (**c**) of dental materials: Enamel plus Hri (I), Amaris (II), and Estelite Asteria (III).

**Figure 7:**
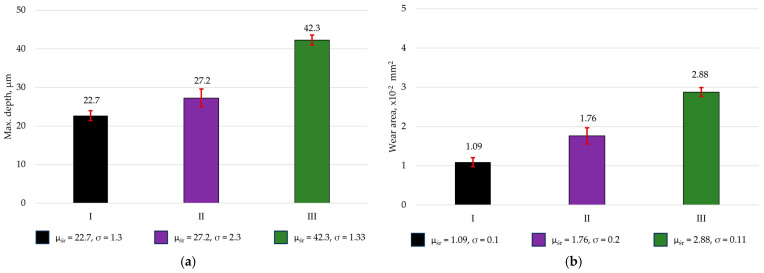
Average value of the maximum depth (**a**) and surface area (**b**) of the wear track of dental materials: Enamel plus Hri (I), Amaris (II), and Estelite Asteria (III).

After the tribological tests, wear tracks were examined (Figure 6 and Figure 7). Based on these observations, the average value of the maximum wear track depth and area was determined (read area). The width of the wear track used for area determination is indicated by a white arrow in the figures.

The results of the wear tests revealed distinct performance differences among the analyzed dental composites, which is consistent with previous reports indicating that the wear behavior of dental resin composites strongly depends on the polymer matrix and mechanical properties of the material. The lowest wear values were observed for Enamel Plus HRi, which may be attributed to its relatively homogeneous structure and the lower proportion of hard, brittle filler particles [35]. Such a microstructure likely promotes a more uniform wear process and reduces the tendency for filler detachment, thereby limiting the formation of third-body abrasive particles within the contact interface. In contrast, the highest wear was recorded for Estelite Asteria, despite it exhibiting the highest instrumented hardness. This finding suggests that, in this material, the dominant wear mechanism is governed by the detachment of hard supra-nano zirconia–silica fillers [28]. Once released, these particles can act as abrasive bodies, scratching the surface and accelerating material loss; this mechanism, in which detached filler particles function as third-body abrasives, has been previously reported for dental resin composites and can outweigh the protective influence typically associated with higher hardness [28].

The results also indicate that materials with more complex surface morphologies exhibited lower friction coefficients due to improved lubricant retention; however, this effect did not translate into reduced wear. This highlights that the friction coefficient alone is not a reliable predictor of wear resistance in dental composites. Wear behavior is more strongly dependent on factors such as filler particle geometry, the quality of filler–matrix bonding, and the stability of this interface under load. The findings demonstrate that composites containing a higher proportion of hard filler particles tend to exhibit increased wear intensity, particularly when filler–matrix adhesion is insufficient to prevent particle dislodgement during tribological loading.

## 4. Conclusions

The preliminary model tests demonstrated the influence of filler composition, morphology, and content on the mechanical, tribological, and wetting properties of the analyzed dental composites. Amaris (II) and Estelite Asteria (III) exhibited the most complex surface morphology, resulting from the presence of large filler particles with diverse structures, as confirmed by SEM observations. The highest hardness—exceeding 300 MPa—was recorded for Estelite Asteria (III), which contains a very high amount of hard silica–zirconia filler particles. In contrast, the highest Young’s modulus was noted for Enamel Plus HRi (I), whose nanohybrid structure provides the greatest stiffness. Amaris, characterized by a moderate filler content, showed the lowest values of instrumented hardness and elastic modulus, amounting to 215 MPa and 8.9 MPa, respectively.

The wettability analysis showed that more developed surface morphology contributes to lowering the contact angle, as observed for the Amaris (II) sample. The remaining materials exhibited hydrophobic behavior, which is clinically desirable because it may reduce the risk of microleakage, bacterial colonization, and staining at the restoration–tooth interface. Tribological tests also revealed that the lowest friction coefficients—0.85—were observed for materials with more complex surface morphology, likely due to improved retention of artificial saliva in micro-indentations. However, this effect did not translate into reduced wear. The smallest wear track area was recorded for Enamel Plus HRi—1.09 × 10^−2^ mm^2^—whose homogeneous structure limited filler detachment, which could otherwise intensify the wear process. In contrast, Estelite Asteria exhibited the highest wear despite its superior hardness, suggesting that its dominant wear mechanism involves the detachment of hard silica–zirconia particles, which subsequently act as abrasive grains. The wear was approximately 62% higher compared to Enamel Plus HRi (I).

It is important to note that the presented results are preliminary and model-based and therefore do not fully reflect the complex conditions of the oral environment. In the following stages of the research, the specimens will undergo various finishing procedures, such as polishing with burs or rubber points. The resulting data will allow assessment of how finishing techniques influence the mechanical, tribological, and functional properties of dental composites, enabling a more comprehensive evaluation of their clinical applicability.

## Figures and Tables

**Table 1:** Characteristics of the materials used for testing.

Name (Manufacturer, Country)	Designation in the Article	Composition	Layers/ Colors	Sample View
Enamel plus Hri, (Micerium S.p.A, Milano, Italy)	I	nano-hybrid composite	4 layers of shade UD2 and the top one—UE2	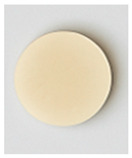
Amaris, (VOCO GmbH, Cuxhaven, Germany)	II	microhybrid conventional composite	4 layers of shade O2 and the top one—TN	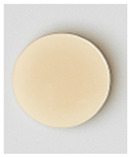
Estelite Asteria, (Tokuyama Dental, Metelen, Germany)	III	supra-nano filled composite	4 layers of shade A2B and the top one—NE	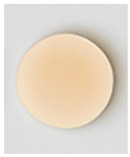

**Table 2:** Resin matrix, filler content, and filler characteristics of the dental composites used in this study.

Name	Resin Matrix (Monomers)	Filler Content, wt%	Filler Type and Size
Enamel plus Hri (I)	UDMA; Bis-GMA; 1,4-butanediol dimethacrylate	75	Dentin shade UD2: glass filler (0.7 µm) + colloidal silica (0.04 µm) Enamel shade UE2: glass filler (1.0 µm) + ZrO_2_ nanoparticles (20 nm)
		80	
Amaris (II)	UDMA, Bis-GMA, TEGDMA	80	Barium–aluminum–boron–silicate glass; methacrylated silica; pyrogenic SiO_2_, Not specified in the manufacturer’s datasheet.
Estelite Asteria (III)	Bis-GMA, Bis-MPEPP, UDMA, TEGDMA	82	Spherical supra-nano zirconia–silica fillers (100–300 nm)

**Table 3:** Chemical composition of the lubricant—artificial saliva [26].

Artificial Saliva, g/dm^3^					
NaCl	KCl	CaCl_2_ ∗ 2H_2_O	NaH_2_PO_4_ ∗ 2H_2_O	Na_2_S ∗ 9H_2_O	Urea
0.4	0.4	0.795	0.780	0.005	1.0

**Table 4:** Hardness measurement results.

Parameter		Enamel Plus Hri (I)	Amaris (II)	Estelite Asteria (III)
Instrumental hardness (H_IT_), MPa	mean	245.8	215.6	300.2
	std. dev.	18.9	8.1	41.9
Young’s modulus (E_IT_), GPa	mean	14.5	8.9	12.7
	std. dev.	0.3	0.7	3.2
Penetration depth (h_m_), nm	mean	1672	2062	1668
	std. dev.	65	45	26

## Data Availability

The original contributions presented in this study are included in the article. Further inquiries can be directed to the corresponding author.
